# AAV expressing an mTOR‐inhibiting siRNA exhibits therapeutic potential in retinal vascular disorders by preserving endothelial integrity

**DOI:** 10.1002/2211-5463.13281

**Published:** 2021-10-21

**Authors:** Seho Cha, Won‐il Seo, Ha‐Na Woo, Hee Jong Kim, Steven Hyun Seung Lee, Jin Kim, Jun‐Sub Choi, Keerang Park, Joo Yong Lee, Beom Jun Lee, Heuiran Lee

**Affiliations:** ^1^ CuroGene Life Sciences Co., Ltd. Cheongju Korea; ^2^ Department of Veterinary Medicine: College of Veterinary Medicine Chungbuk National University Cheongju Korea; ^3^ Department of Microbiology College of Medicine University of Ulsan Seoul Korea; ^4^ Bio‐Medical Institute of Technology College of Medicine University of Ulsan Seoul Korea; ^5^ Department of Medical Science Asan Medical Institute of Convergence Science and Technology Asan Medical Center University of Ulsan College of Medicine Seoul Korea; ^6^ Department of Ophthalmology Asan Medical Center University of Ulsan College of Medicine Seoul Republic of Korea; ^7^ Department of Microbiology Asan Medical Center University of Ulsan College of Medicine Seoul Republic of Korea

**Keywords:** angiogenesis, adeno‐associated virus, endothelial cells, migration, proliferation, retinal vascular disorder, small‐hairpin mTOR, tight junction

## Abstract

Expanding on previous demonstrations of the therapeutic effects of adeno‐associated virus (AAV) carrying small‐hairpin RNA (shRNA) in downregulating the mechanistic target of rapamycin (mTOR) in *in vivo* retinal vascular disorders, vascular endothelial growth factor (VEGF)‐stimulated endothelial cells were treated with AAV2‐shmTOR to examine the role of mTOR inhibition in retinal angiogenesis. AAV2‐shmTOR exposure significantly reduced mTOR expression in human umbilical vein endothelial cells (HUVECs) and decreased downstream signaling cascades of mTOR complex 1 (mTORC1) and mTORC2 under VEGF treatment. Moreover, the angiogenic potential of VEGF was significantly inhibited by AAV2‐shmTOR, which preserved endothelial integrity by maintaining tight junctions between HUVECs. These data thus support previous *in vivo* studies and provide evidence that AAV2‐shmTOR induces therapeutic effects by inhibiting the neovascularization of endothelial cells.

## Graphical abstract

Inhibition of mTOR expression by AAV2‐shmTOR suppresses the proliferation and migration of endothelial cells stimulated by VEGF. mTOR is a major activator in endothelial cells under VEGF stimulation, and inhibition by AAV2‐shmTOR blocked the activation of downstream mTOR signaling molecules. Inhibition of the mTOR pathway consequently affected cell proliferation and the remodeling of tight junctions between endothelial cells.

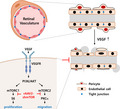

**Abbreviations**
AAV: adeno‐associated virus
AMD: age‐related macular degeneration
CNV: choroidal neovascularization
HUVEC: human umbilical vein endothelial cell
mTOR: mechanistic target of rapamycin
mTORC1: mTOR complex 1
mTORC2: mTOR complex 2
OIR: oxygen‐induced retinopathy
shRNA: small‐hairpin RNA
VEGF: vascular endothelial growth factor

Neovascularization is one of the most common features of retinal vascular disorders, including exudative age‐related macular degeneration (AMD), diabetic retinopathy (DR), and retinopathy of prematurity. Pathological angiogenesis of endothelial cells in the intraocular blood vessel generally results in irreversible blindness or visual impairment [1, 2]. Various cellular factors, including platelet‐derived growth factor, fibroblast growth factor, and angiopoietin, regulate angiogenesis [3]. Among these factors, vascular endothelial growth factor (VEGF) is known as the most critical regulator of angiogenesis [4]. There have been extensive studies on VEGF‐mediated intracellular signaling cascades and its roles in angiogenic properties [3, 5]. These data suggested an anti‐VEGF strategy as a promising therapeutic approach for degenerative retinal disorders [6]. Some anti‐VEGF drugs were approved and used in treating retinal neovascular disorders [7, 8, 9]. However, there are several aspects to be considered to apply anti‐VEGF therapeutic drugs for retinopathy. First, because VEGF is an upstream regulator in a variety of cellular signaling cascades, including cell survival/apoptosis and inflammatory responses, the inhibition of VEGF may dysregulate the maintenance of normal intracellular condition. Second, some patients with AMD or diabetic macular edema exhibited recurrent or refractory symptoms after being treated with standard anti‐VEGF therapy. Third, traditional anti‐VEGF therapy requires repeated intravitreal administration of agents monthly. Considering the role of VEGF in the homeostasis of the ophthalmic system and the unexpected adverse effects, there exists a need to develop novel strategies such as other anti‐VEGF drugs, combination therapy, and multitarget treatments [10, 11, 12].

The mechanistic target of rapamycin (mTOR) is a phosphoinositide 3‐kinase (PI3K)‐related protein kinase and largely exists as a constituent component of two complexes, mTOR complex 1 (mTORC1) and mTORC2. Although several differences exist in the components and downstream effectors, the functions of these two complexes are intertwined with various functions in cell growth and proliferation [13]. mTOR signaling is largely involved in the angiogenic activity of VEGF. Inhibition of mTOR by rapamycin could efficiently reduce metastatic tumor growth and angiogenic properties, accompanied by a reduction in the mRNA level of VEGF [14]. In the ophthalmic system, studies have reported that VEGF activates the mTOR‐dependent pathway in the proliferation of endothelial cells of neonatal mouse retina, and inhibition of mTOR partially reduced vascularization and capillary density during early development (postnatal day 0 to P7) [15, 16]. It has also been reported that a dramatic increment of mTOR activation occurs in a damaged retina [17].

In our previous studies, we demonstrated that inhibition of the mTOR signaling pathway using a small‐hairpin RNA (shRNA) delivered via recombinant adeno‐associated virus 2 (AAV2) (termed rAAV2‐shmTOR‐SD, renamed here as AAV2‐shmTOR) led to an effective reduction of neovascularization in the mouse choroidal neovascularization (CNV) and rat oxygen‐induced retinopathy (OIR) models [18, 19, 20]. In those results, we confirmed the successful transduction of AAV pseudotyped with serotype AAV2 on the retinal tissues. The inhibition of mTOR had functional roles in anti‐angiogenesis, anti‐inflammatory responses, and anti‐proliferation in those animal models. However, there are several aspects of AAV2‐shmTOR functions during angiogenic events that need to be explored at the molecular level.

In the present study, we examined the intracellular and intercellular responses to the antiangiogenic activity of mTOR inhibition by AAV2‐shmTOR treatment in the VEGF‐stimulated state of endothelial cells. We found that AAV2‐shmTOR significantly reduced the mTOR expression and downstream signaling cascades of mTORC1 and mTORC2 in HUVECs, even under VEGF treatment conditions. AAV2‐shmTOR efficiently inhibits the angiogenic potential of VEGF‐stimulated HUVECs. Furthermore, the endothelial integrity of HUVECs was supported by AAV2‐shmTOR, which sustained the tight junctions between cells. These observations indicate that AAV2‐shmTOR could be a major therapeutic for retinal vascular disorders.

## Materials and methods

### Cell culture and reagents

HUVECs were commercially purchased from LONZA (Basel, Switzerland). The cells were cultured in endothelial cell growth medium‐2 (EGM™‐2, LONZA) supplemented with EGM‐2 Single Quots^TM^ (LONZA) and maintained in a humidified atmosphere of 5% CO_2_ at 37 °C in an incubator. To synchronize the cells before VEGF treatment, HUVECs were incubated in endothelial cell basal medium‐2 (EBM™‐2, LONZA) supplemented with 2% fetal bovine serum (Cytiva, MA, USA). Recombinant human VEGF_165_ was purchased from R&D Systems (MN, USA).

### Preparation of rAAV2

The virus vectors used for producing AAV2‐shmTOR and AAV2‐shCon were prepared as previously described [18, 19]. The shRNA sequence 5′‐GAAUGUUGACCAAUGCUAU‐3′ for inhibiting mTOR or the control shRNA sequence 5′‐AUUCUAUCACUAGCGUGAC‐3′, with H1 promoter, was cloned into scAAV2‐GFP vector, and an expression cassette for EGFP was replaced by human UBE3A gene fragment (accession no. AH006486.2, position 994 to 2740). AAV2 was produced using a triple co‐transfection method as described previously [19].

### Animal care and laser‐induced CNV model

Male C57BL/6 mice aged 8 weeks were purchased from The Orient Bio (Sungnam, Korea). All animal care procedures and experiments were conducted according to the guidelines stated in the Association for Research in Vision and Ophthalmology Resolution on the Use of Animals in Ophthalmic and Vision Research. The Internal Review Board approved the study for Animal Experiments at the Asan Institute for Life Science (University of Ulsan, College of Medicine). Mice were anesthetized by an intraperitoneal injection of a mixture of Zoletil (40 mg·kg^−1^ zolazepam and tiletamine) purchased from Virbac (Carros Cedex, France) and Rompun (5 mg·kg^−1^ xylazine) purchased from Bayer Healthcare (Leverkusen, Germany), and their pupils were dilated using Mydrin‐P (0.5% tropicamide and 2.5% phenylephrine) acquired from Santen (Osaka, Japan). A 532‐nm neodymium‐doped yttrium aluminum garnet PASCAL diode ophthalmic laser system (Topcon Medical Laser Systems, Santa Clara, CA, USA) was used for laser photocoagulation (200 µm spot size, 0.02 s duration, 100 mW), wherein five or six laser spots were applied around the optic nerve of only the right eye. Gaseous bubble formation at the laser spot indicated the rupture of Bruch’s membrane.

### Wound healing assay

For wound healing assay, HUVECs were seeded at a density of 4000 cells/insert on a silicon insert (Ibidi, Bavaria, Germany) in 6‐well plates. After reaching > 90% confluence, the silicon insert was removed, and HUVECs were incubated in EBM™‐2 supplemented with 2% FBS in the presence or absence of 10 ng·mL^−1^ VEGF. For rAAV2 treatment, AAV2‐shmTOR or AAV2‐shCon was infected at 200 000 MOI at 24 h before VEGF treatment. The gap closure was analyzed at the indicated time points. The area of the remaining gap region was calculated using the imagej MRI wound healing tool.

### Tube formation assay

After incubating AAV2‐shmTOR or AAV2‐shCon for 24 h (Cell Biolabs, Inc., San Diego, CA), 3 × 10^4^ HUVECs were seeded on ECM gel in 96‐well plates either in the presence or in the absence of VEGF for 1 h. The tube‐forming activity of HUVECs was observed within 4 h. The nodes of tubular structures were quantified using imagej software.

### Permeability assay

Cellular permeability was analyzed using In Vitro Vascular Permeability Imaging Assay (Merck, #17‐10398, Temecula, CA). 8‐well chamber slides were sequentially treated with poly‐L‐Lysine, glutaraldehyde, and biotinylated‐gelatin. Coated slides were disinfected with 70% ethanol and then washed with DPBS. Residual‐free aldehydes were quenched by washing the chamber slides using a growth medium. 1.5 × 10^4^ HUVECs were seeded on the coated chamber slides and treated with AAV2‐shmTOR or AAV2‐shCon for 24 h. After incubation of VEGF for 1 h, fixation and staining were performed according to the manufacturer’s instructions. Images were observed with a confocal microscope and quantified using imagej software.

### Proliferation assay

HUVECs were seeded at a density of 4000 cells·well^−1^ in 96‐well plates. AAV2 or inhibitor was pretreated at 16 and 1 h before VEGF (in EBM™‐2 supplemented with 2% FBS) treatment, respectively. At the indicated time points after VEGF treatment, a mixture of CCK‐8 (10 μL of CCK‐8 and 100 μL of EBM™‐2 supplemented with 2% FBS) was added into each well. After incubation for 1 h, the absorbance was measured using a microplate spectrophotometer (Epoch, BioTek, VT, USA) and normalized by a control value.

### Western blotting

HUVECs were seeded on 12‐ or 6‐well plates at a density of 40 000 or 200 000 cells·well^−1^, respectively. After 24 h, HUVECs were synchronized with EBM™‐2 supplemented with 2% FBS for 24 h. Cells were then lysed with RIPA buffer (ELPIS‐BIOCH, Daejeon, Korea) containing phosphatase inhibitor cocktail 3 (Sigma, MO, USA) at 5 min or the indicated time points after VEGF treatment. Protein concentration was determined using the Pierce™ BCA Protein Assay Kit (Thermo Fisher Scientific, MA), and an equal concentration of protein was loaded for western blotting analysis. The following primary antibodies were used in this study: mTOR (#2983, Cell Signaling, Danvers, MA), p‐PKCα S657 (ab180848, Abcam, Boston, MA), PKCα (sc‐8393, Santa Cruz, Santa Cruz, CA), p‐S6 S235 (ab12864, Abcam), S6 (sc‐74459, Santa Cruz), β‐actin (sc‐47778, Santa Cruz), and ZO‐1 (ab96587, Abcam). The primary antibodies were then bound to peroxidase‐conjugated secondary antibodies diluted in blocking solution, and their binding on the membrane was detected using enhanced chemiluminescence (Luminograph II, ATTO, Tokyo, Japan).

### Immunofluorescence assay

For staining the tight junction protein ZO‐1, HUVECs were seeded at a density of 300 000 cell·well^−1^ in 6‐well plates. After 24 h of AAV2 infection, HUVECs were synchronized with EBM™‐2 supplemented with 2% FBS for 24 h. After 30 min of VEGF treatment, HUVECs were fixed with acetone:methanol (1 : 1) on ice for 10 min. After washing with PBS and blocking with 1% BSA in PBST (0.1% Tween 20) for 30 min, HUVECs were incubated with the anti‐ZO‐1 antibody at 4 °C for 16 h. The cells were again washed with PBS and incubated with anti‐rabbit IgG‐FITC for 1 h. Then, HUVECs were mounted with the VECTASHIELD® DAPI solution (Vector Laboratories, CA, USA), and images were obtained using a Nikon Ts2 FL Microscope (Nikon, Tokyo, Japan).

### Statistical analysis

Results were statistically analyzed using Student’s *t*‐test or ANOVA, followed by post hoc analysis using the Scheffe test. The experiments were performed at least in triplicate. Significant differences were determined at *P < 0.05, **P < 0.01, or ***P < 0.001. Graphs were used to visualize the data and include significance and mean ± SD values.

## Results

### Transduction of endothelial cells by AAV2 vector in the laser‐induced lesions of CNV model

**Fig. 1:**
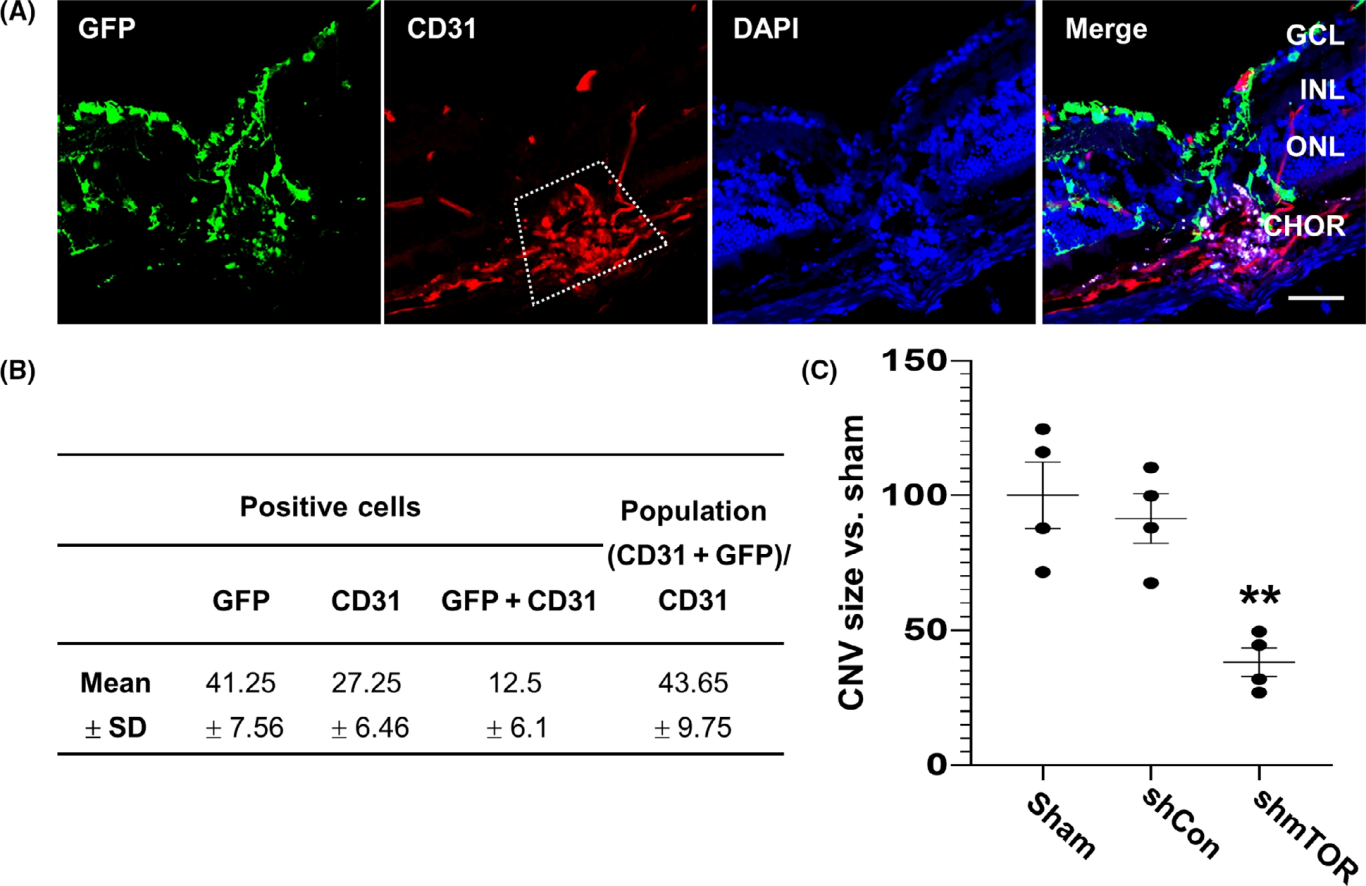
Transduction of endothelial cells by AAV2 vector in the CNV model. (A) AAV2‐GFP was intravitreally injected into the laser‐induced photocoagulated eyes of the mouse. Frozen section samples were stained with anti‐GFP along with anti‐CD31 (red) to determine the retinal tissue tropism of AAV2‐shmTOR. Nuclei were counterstained with DAPI (blue). Box: CNV lesion, Scale bars: 20 µm. (B) The size of the retinal photocoagulation lesion was measured by light microscopy after immunostaining anti‐cytokeratin and anti‐CD31 using a cross‐sectioned eye. (C) The extent of overlap of the GFP signal and red fluorescence for CD31 in the CNV lesion was quantified by the imagej software. All panels are representative of at least three independent experiments (*n* = 4). ANOVA and paired *t*‐test. **P < 0.01.

We had previously confirmed that AAV2‐shmTOR infection efficiently reduced CNV in the mouse model [18]. To explore the mechanism by which AAV2‐shmTOR can affect the laser‐induced lesions, we first investigated the localization of CD31‐expressing endothelial cells and viral‐transduced cells using AAV2‐GFP in the CNV mouse model. We observed an increment of endothelial cells in the CNV model, especially in the area around the laser‐induced lesion. As depicted in Fig. 1A,B, GFP‐expressing cells were colocalized with CD31‐expressing cells (43.65% ± 9.75%), which is consistent with the previous OIR model [20]. We next examined mTOR expression after the transduction of AAV2‐shmTOR in the CNV model and found that mTOR expression was increased in the laser‐induced lesions. However, we observed that mTOR expression in endothelial cells was substantially suppressed by AAV2‐shmTOR transduction in the laser‐induced lesions (Fig. S1) and the size of the CNV lesion was reduced by AAV2‐shmTOR (38.14% ± 10.58%, Fig. 1C). These results indicate successful transduction by the recombinant AAV pseudotyped with serotype AAV2 on retinal endothelial cells, suggesting that the inhibitory role of AAV2‐shmTOR in neovascularization is directly exerted on the intracellular responses of the endothelial cells.

### Regulation of the cellular signaling pathway by AAV2‐shmTOR in endothelial cells

**Fig. 2:**
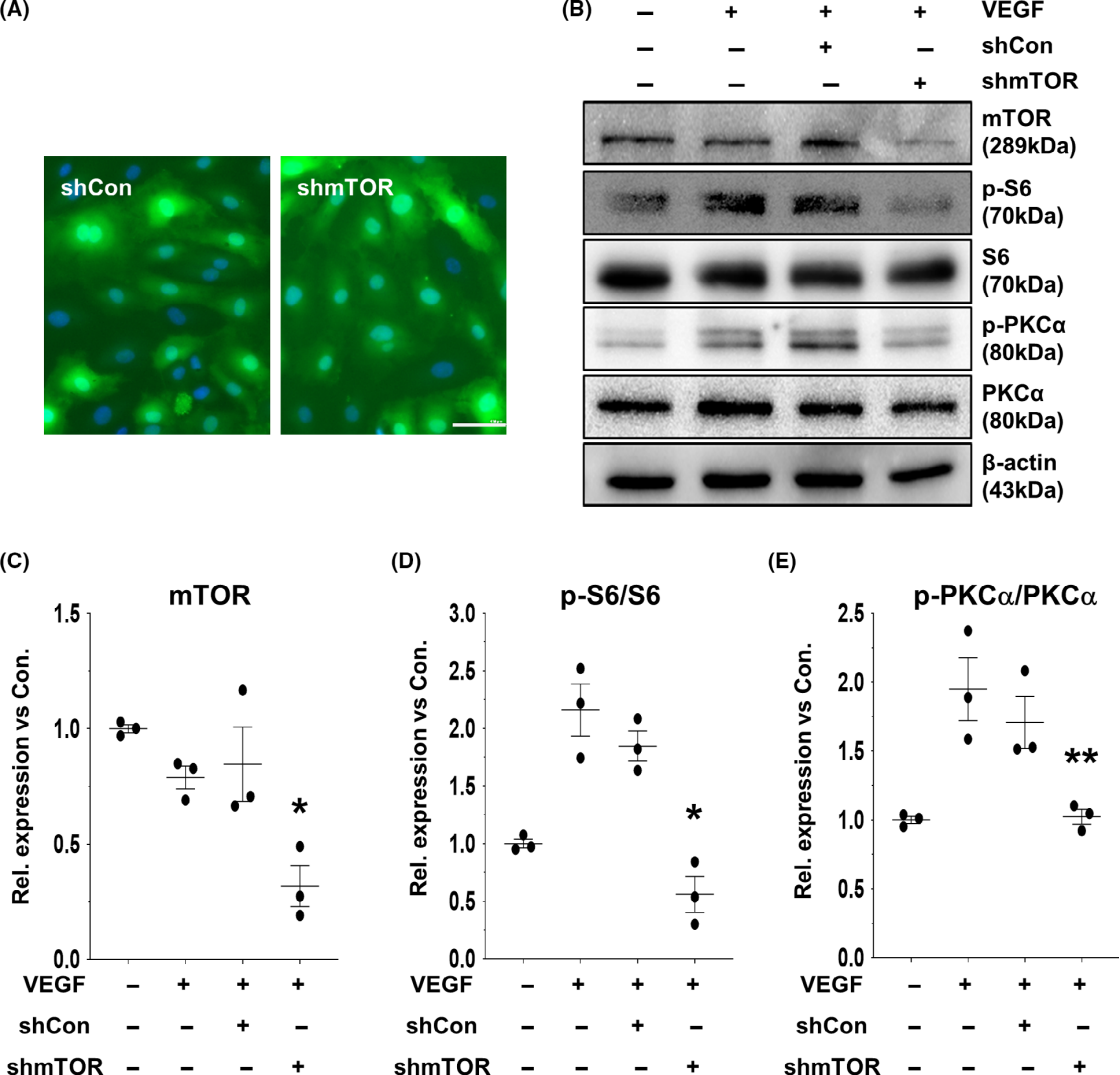
Downregulation of mTOR signaling pathways by AAV2‐shmTOR. (A) HUVECs were transduced by AAV2‐GFP, and the expression of GFP was observed by fluorescence microscopy. Scale bars: 100 µm. (B) Downregulation of the expression level of mTOR and the downstream signaling molecules of mTOR were examined at 24 h after AAV2‐shmTOR infection with cotreatment of VEGF (5 min, 10 ng·mL^−1^) in HUVECs, as indicated. (C–E) Graphs show the relative expression levels of mTOR, p‐PKCα, and p‐S6 versus the total amount of corresponding protein. Mean and standard deviation were presented from three independent experiments (*n* = 3). ANOVA and paired *t*‐test. *P < 0.05, **P < 0.01.

To evaluate the intracellular responses by AAV2‐shmTOR in endothelial cells, we compared the effect of AAV2‐shmTOR with its nonspecific negative control, AAV2‐shCon. Almost the entire transduction of HUVECs by rAAV2 was achieved when cells were treated with AAV2‐GFP (Fig. 2A). Results of western blotting and real‐time RT‐qPCR showed that AAV2‐shmTOR dramatically reduced mTOR expression after infection in HUVECs, whereas the expression level of mTOR was not affected by the transduction of AAV2‐shCon (Fig. 2B, Fig. S2), suggesting that AAV2‐shmTOR could effectively transduce HUVECs and downregulate mTOR expression.

Next, we examined the regulation of mTOR by AAV2‐shmTOR under VEGF‐induced stimulatory condition in HUVECs. To provide sufficient time for expressing the shRNA, HUVECs were infected with AAV2‐shmTOR or AAV2‐shCon 24 h before VEGF treatment. As shown in Figs. 2B and 2C, the expression of mTOR was successfully inhibited by AAV2‐shmTOR in the presence of VEGF and the signaling of mTOR was also suppressed. Consequently, the phosphorylation levels of S6 and PKCα, the respective downstream substrates of mTORC1 and mTORC2, were significantly reduced by AAV2‐shmTOR even in the presence of VEGF, whereas the total amount of protein expressions for PKCα and S6 remained unaffected (Figs. 2B,D,E). These results indicate that AAV2‐shmTOR is potent to suppress mTOR signaling even under the stimulation conditions of endothelial cells.

### Antiangiogenic potential of AAV2‐shmTOR in endothelial cells

**Fig. 3:**
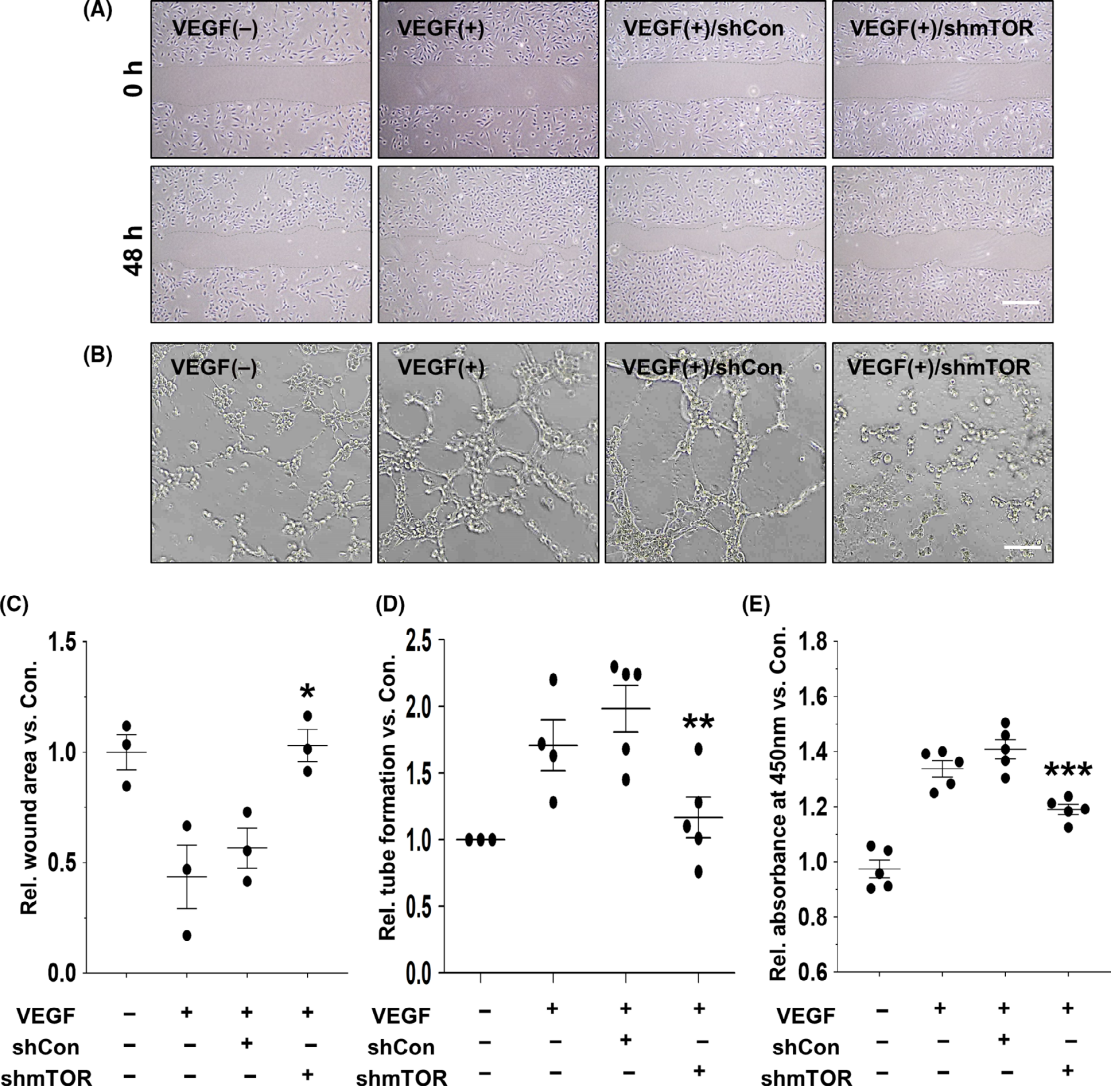
Antiangiogenic properties of AAV2‐shmTOR in endothelial cells. (A, C) 48 h after VEGF treatment, the migration of HUVECs transduced with AAV2‐shCon or AAV2‐shmTOR was analyzed by the wound healing assay. Dotted lines indicate the boundaries of migrating cells. The graph presents the mean values with standard deviations from three independent experiments (*n* = 3). Scale bars: 1 mm. (B, D) Tube‐forming assay observations of HUVECs transduced with AAV2‐shCon or AAV2‐shmTOR for 24 h. Tube‐forming activity of HUVECs was observed within 4 h after VEGF treatment. The nodes of tubular structures were quantified (*n* ≥ 3). Scale bars: 100 μm. (E) Proliferation of HUVECs treated with AAV2‐shCon of AAV2‐shmTOR was examined by CCK‐8 assay in the presence of VEGF stimulation. The relative absorbance at 450 nm was presented as mean and standard deviation from at least three independent experiments (*n* = 5). ANOVA and paired *t*‐test. *P < 0.05, **P < 0.01, ***P < 0.001.

To evaluate whether AAV2‐shmTOR exhibits antiangiogenic potential in endothelial cells under angiogenic stimulation, we examined the migration of HUVECs after VEGF treatment using the wound healing assay. The HUVECs treated with AAV2‐shmTOR were observed to maintain the wounded area until 48 h after VEGF treatment, whereas mock‐treated HUVECs and the AAV2‐shCon group displayed markedly decreased wound area caused by active migration (Figs. 3A,C). In the tube‐forming assay, AAV2‐shmTOR demonstrated effective suppression of HUVECs’ tubular structures induced by VEGF stimulation (1.17 ± 0.34 fold‐induction; Figs. 3B,D). We next explored the antiproliferative function of AAV2‐shmTOR in endothelial cells using the CCK‐8 assay. Results showed that VEGF treatment induced the proliferation of HUVECs (1.45 ± 0.08 fold‐induction) in mock‐treated and AAV2‐shCon groups. HUVECs in the AAV2‐shmTOR group maintained a normal level of proliferation (Fig. 3E). These results indicate that the downregulation of mTOR by AAV2‐shmTOR is effective in suppressing the abnormal neovascularization activity of endothelial cells by VEGF treatment.

### Effects of AAV2‐shmTOR in the maintenance of endothelial integrity

**Fig. 4:**
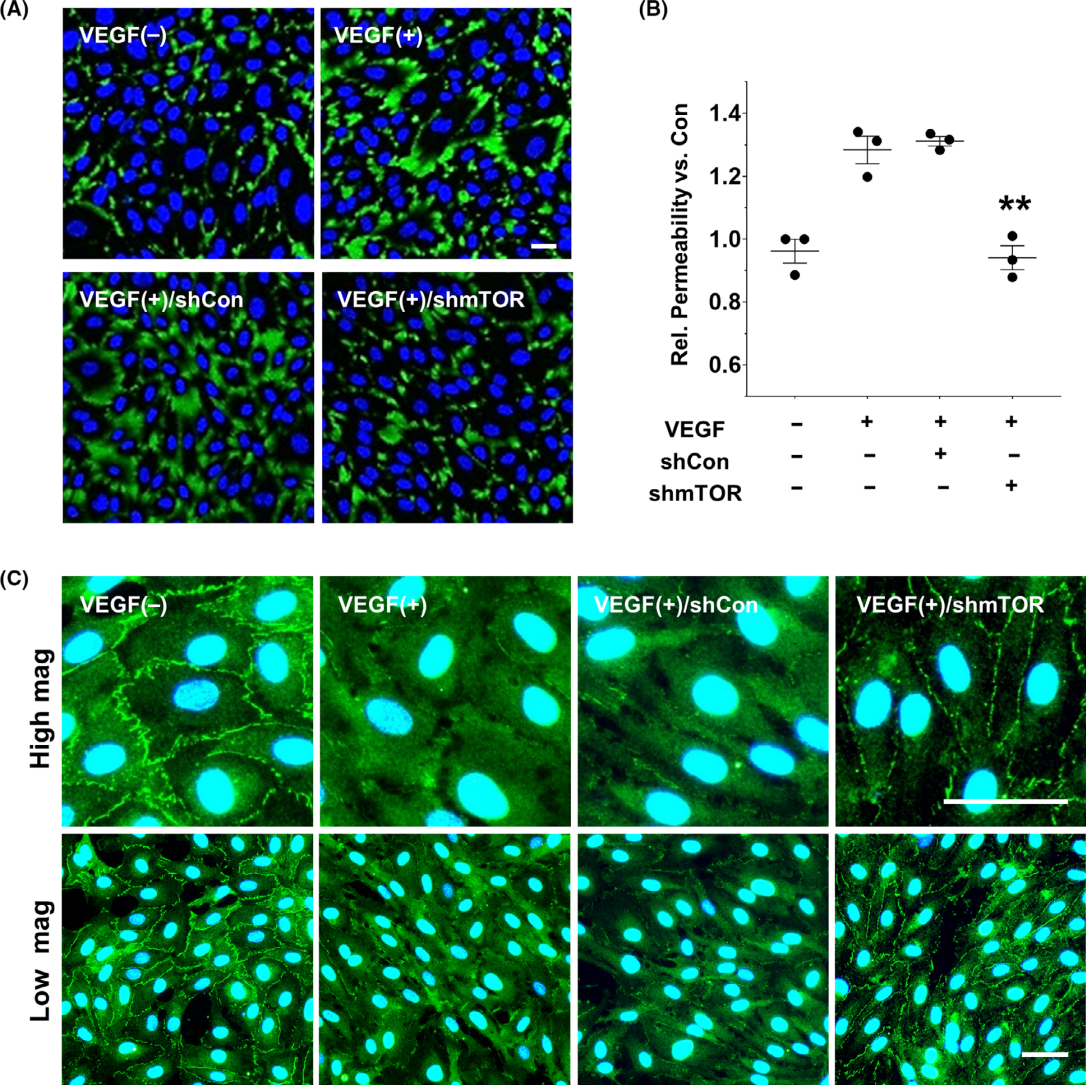
Preventative effects of AAV2‐shmTOR on VEGF‐mediated membrane dysfunction. (A, B) VEGF‐treated HUVECs’ cell permeability was observed via imaging analysis. AAV2‐shmTOR inhibited cell barrier disruptions caused by VEGF (*n* = 3). (C) Cellular localization of the tight junction protein ZO‐1 was analyzed by immunostaining in HUVECs. Downregulation of mTOR by AAV2‐shmTOR prevents the dysregulation of ZO‐1 localization, whereas VEGF treatment induced the disruption of ZO‐1 localization in the plasma membrane fractions. Scale bars: 50 µm. ANOVA and paired *t*‐test. **P < 0.01.

Vascular endothelial cell barriers were examined to further determine the specific downstream effector of AAV2‐shmTOR on VEGF‐stimulated anti‐neovascularization activity. Transepithelial resistance was analyzed using fluorescence‐based permeability imaging assay. VEGF substantially increased cellular permeability (1.28 ± 0.08 fold‐induction), whereas HUVECs transduced by AAV2‐shmTOR maintained endothelial integrity (0.94 ± 0.07 fold‐induction; Figs. 4A,B). In addition, we analyzed the intracellular location of a tight junction protein, zonula occludens‐1 (ZO‐1). Immunofluorescence assay of HUVECs revealed that ZO‐1 was localized at the tight junction between cells. However, ZO‐1 at the cell–cell contact sites disappeared rapidly within 30 min after VEGF treatment in HUVECs (Fig. 4C). In HUVECs transduced by AAV2‐shmTOR, ZO‐1 was retained at the contact sites between cells similar to that under normal condition (Fig. 4C). We also confirmed whether the distribution of ZO‐1 in the mTOR inhibitor rapamycin‐treated HUVECs was similar to that in AAV2‐shmTOR‐treated HUVECs (data not shown). Altogether, these results suggest that the silencing of mTOR using AAV2‐shmTOR bolsters endothelial integrity by maintaining tight junctions in endothelial cells.

**Fig. 5:**
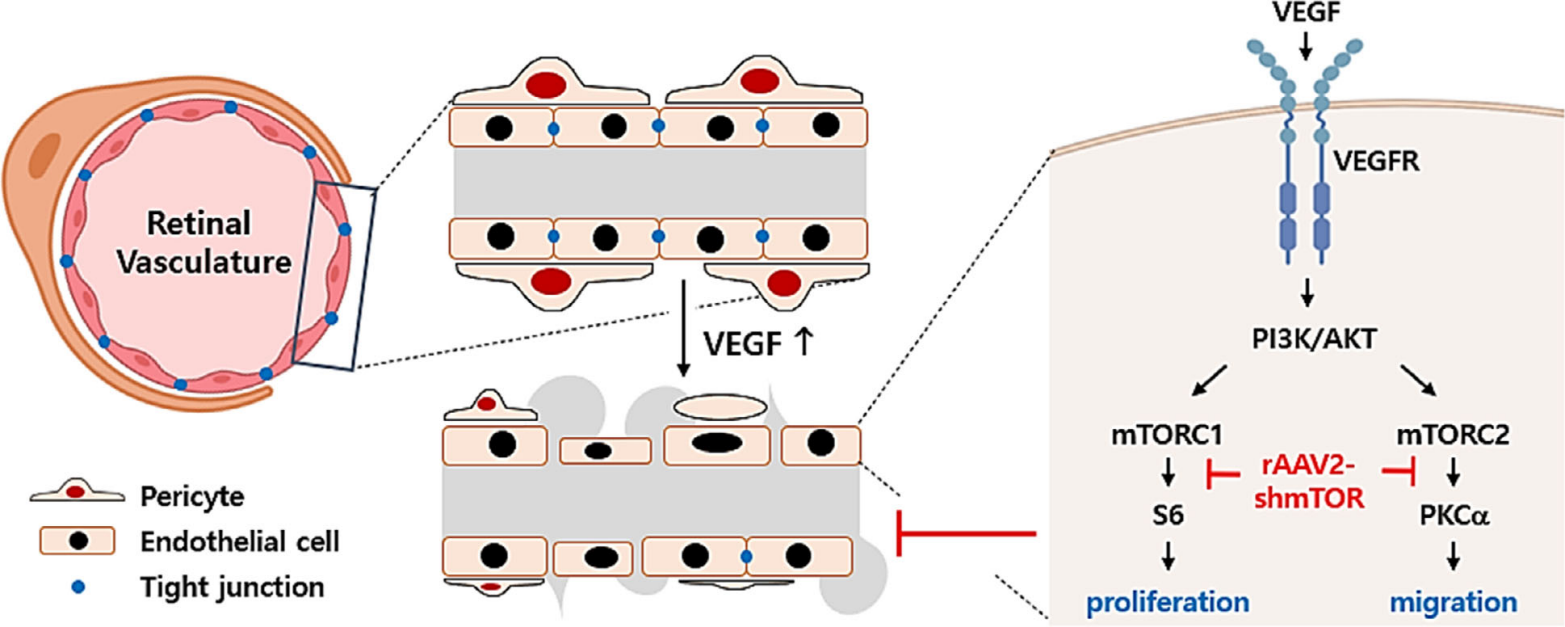
Schematic diagram of the function of AAV2‐shmTOR. Inhibition of mTOR expression by AAV2‐shmTOR suppresses the proliferation and migration of endothelial cells stimulated by VEGF. mTOR is a major activator in endothelial cells under VEGF stimulation, and inhibition of mTOR by AAV2‐shmTOR blocked the activation of downstream signaling molecules such as S6 and PKCα. Consequently, inhibition of the mTOR pathway affected the cell proliferation and remodeling of the tight junction between endothelial cells.

Fig. 5 illustrates the mechanism of anti‐angiogenesis activity of AAV2‐shmTOR based on the above‐described experiments. Upon abnormal neovascularization stimuli, mTOR is activated and plays a role as a driving force for proliferation and migration. In this pathological condition, the downregulation of mTOR level by AAV2‐shmTOR was found to be sufficient to block the abnormal behavior of endothelial cells.

## Discussion

This study provides evidence at the cellular level that the inhibition of mTOR by AAV2‐shmTOR induced antiproliferative as well as antiangiogenic effects in endothelial cells upon VEGF stimulation. These results support our previous findings that suggested an effective reduction of neovascularization by AAV2‐shmTOR in CNV and OIR models [8, 20]. Retinal vascular diseases share salient and common features of retinal neovascularization. VEGF is known as a major driver of abnormal proliferation of retinal endothelial cells. Therefore, anti‐VEGF therapeutic strategies have been followed and approved to treat these conditions. However, previous studies have suggested that sustained VEGF inhibition could cause considerable problems in eyes [8, 21], thereby raising a further need to develop novel therapeutic strategies beyond conventional anti‐VEGF therapies.

Due to its role as a major modulator of cell growth and proliferation, inhibition of mTOR has been considered as an important drug development target for cancers. Several chemical drugs such as rapalogs, derivatives of mTOR inhibitor rapamycin, have been approved for treating renal cell carcinoma, osteosarcoma, and breast cancers [22, 23, 24]. In addition to cancer cells, mTOR activation is essential for various types of cells, and an irregular activation of mTOR was found to be closely related to diseases caused by excessive cell growth and proliferation. Recent studies have proposed mTOR inhibition as a novel therapeutic strategy for neovascularization‐related ocular disorders based on the antiproliferative and antiangiogenic activity of the suppression of mTOR. One of such studies reported that the PI3K/mTOR inhibitor GNE‐947 reduced the area of laser‐induced CNV lesions in rat models [25]. The authors showed that targeting PI3K/mTOR suppressed the angiogenesis stimulated by VEGF. Another study demonstrated activation of the mTOR signaling pathway during CNV pathogenesis, especially in infiltrated inflammatory cells [26]. In the present study, mTOR inhibition was associated with low expression levels of antiangiogenic regulators. These results indicate that the inhibition of mTOR may be a significant strategy for the development of antiangiogenic agents in treating retinal vascular disorders causing new vessel formation. Currently, there is no approved therapeutic drug that can directly target mTOR for treating ocular disorders.

Therefore, we explored the function of AAV2‐shmTOR in VEGF‐stimulated endothelial cells based on the results of colocalization of CD31‐positive endothelial cells with GFP‐expressing cells by AAV2‐GFP in the retinal tissues of the CNV model (Fig. 1). We observed that downregulation of mTOR by the transduction of AAV2‐shmTOR had an inhibitory effect on angiogenic potential of HUVECs upon VEGF stimulation by blocking the activation of both mTORC1 and mTORC2 (Figs. 2,3). Phosphorylation of the downstream substrates of mTORC1 and mTORC2, S6 and PKCα, was significantly reduced by AAV2‐shmTOR transduction (Fig. 2). Activation of the mTOR pathway by VEGF contributes to the angiogenic potential of endothelial cells through the activation of these substrates. mTORC1 phosphorylates and activates S6 kinase and activates S6 kinase‐phosphorylated S6 ribosomal protein. The phosphorylated S6 protein can regulate the translation of mRNA to support the proliferation of cells. mTORC2 activates PKCα, and the activity of PKCα is known to be related to cell adhesion and migration by inducing actin cytoskeleton changes [27, 28].

The retina is a highly differentiated tissue with complex multilayered structures consisting of various cells with specialized functions such as rods, cones, Müller cells, retinal endothelial cells, and retinal pigment epithelial cells (RPE). To retain a constant milieu, retinal capillary endothelial cells and RPE cells maintain the blood–retina barrier (BRB) by intercellular junctions. In fact, VEGF is one of the most potent inducers of vascular permeability on the endothelium. We confirmed the preventive role of AAV2‐shmTOR in the loss of transepithelial resistance caused by VEGF (Figs. 4A,B). The function of AAV2‐shmTOR on the maintenance of endothelial integrity was also explored. Observations showed that VEGF stimulation induces a dysregulation of ZO‐1 localization in confluent HUVECs and that treatment with AAV2‐shmTOR restores ZO‐1 localization in intercell junctions (Fig. 4C). The disruption of the tight junctions by VEGF was prevented by pretreatment with the pan‐PKC inhibitor Gö6983 (data not shown). Our findings indicated that the activation of PKCα by mTOR after VEGF stimulation directly participates in the disruption of tight junctions in HUVECs. AAV2‐shmTOR can exhibit antiangiogenic activity by maintaining the tight junctions between endothelial cells under the neovascularization stimuli in animal neovascularization‐associated models.

Consistent with other studies, we have previously confirmed the effective role of the inhibition of mTOR in anti‐angiogenesis in animal neovascularization‐associated models [19, 20]. Considering the safety and long‐term efficacy of AAVs, a useful strategy in the treatment of human diseases, we developed the AAV2 vector to deliver shRNA for mTOR. We found that the intravitreal administration of AAV2‐shmTOR could transduce throughout the retinal compartment, including retinal endothelial cells [18]. Because mTOR is known to exert different roles in various cell types [13], there is a need to evaluate the specific roles of AAV2‐shmTOR in the retinal endothelial cells. Furthermore, in‐depth studies are required to improve our understanding about the therapeutic effects of AAV2‐shmTOR in animal models of retinal diseases. As mentioned earlier, studies on a single type of cells have not been adequate to comprehend the physiological events in retinal disorders because various cells interact and influence each other in the retina. We are currently planning to investigate the therapeutic activity of AAV2‐shmTOR in 3D culture models. We shall also examine the therapeutic benefits of combination therapy for mTOR and VEGF targeting for retinal neovascularization diseases.

In conclusion, we elucidate the antiproliferative and anti‐angiogenic mechanisms of AAV2‐shmTOR in endothelial cells. Inhibition of the mTOR signaling cascade is sufficient to reduce VEGF‐mediated angiogenic responses. We also suggest that the maintenance of cell–cell interaction by preventing the VEGF‐mediated disruption of tight junctions can contribute to the antiangiogenic role of AAV2‐shmTOR. Our study findings provide evidence for the therapeutic potential of AAV2‐shmTOR in treating retinal neovascular‐associated disorders.

## Conflict of interest

SC, HJK, SHSL, JK, J‐SC, and KP are employees of CuroGene Life Sciences Co., Ltd., in which SHSL has a personal financial interest. No other potential conflicts of interest relevant to this article were reported.

## Author contributions

SC, W‐IS, H‐NW, J‐SC, KP, JYL, BJL, and HL conceptualized the study, for which SC, W‐IS, H‐NW, HJK, JK, J‐SC, BJL, and HL developed the methodology. SC, W‐IS, H‐NW, J‐SC, KP, JYL, BJL, and HL performed investigation. SC, H‐NW, SHSL, and J‐SC wrote the original draft of the article, which was reviewed and edited by SHSL, KP, JYL, BJL, and HL.

## Supporting information


**Fig. S1**. Colocalization of mTOR and CD31. Immunostain visualizing that mTOR‐ and CD31‐positive cells were observed in laser‐induced CNV lesions, with mTOR (green) being found in CD31 (red)‐positive cells. Scale bar: 50 μm.
**Fig. S2**. Inhibition of mTOR expression by AAV2‐shmTOR‐SD. mTOR expression, induced by VEGF‐A and detected via real‐time PCR, was observed in HUVEC cells. Data was analyzed via paired *t*‐test, and expressed as normal constant ratio and mean ± SEM. **P < 0.01.

## Data Availability

The data that support the findings of this study are available in the figures and the Supporting information of this article.
