# Supplementary material for: AAV expressing an mTOR‐inhibiting siRNA exhibits therapeutic potential in retinal vascular disorders by preserving endothelial integrity
Source: FEBS Open Bio. 2021 Oct 21;12(1):71–81. doi: 10.1002/2211-5463.13281 (PMC8727948; doi:10.1002/2211-5463.13281)
Supplement: Supplementary file 1 — Fig. S1. Colocalization of mTOR and CD31. Immunostain visualizing that mTOR‐ and CD31‐positive cells were observed in laser‐induced CNV lesions, with mTOR (green) being found in CD31 (red)‐positive cells. Scale bar: 50 μm. Fig. S2. Inhibition of mTOR expression by AAV2‐shmTOR‐SD. mTOR expression, induced by VEGF‐A and detected via real‐time PCR, was observed in HUVEC cells. Data was analyzed via paired t‐test, and expressed as normal constant ratio and mean ± SEM. **P < 0.01. [file FEB4-12-71-s001.pdf]

Supplemental Figure 1

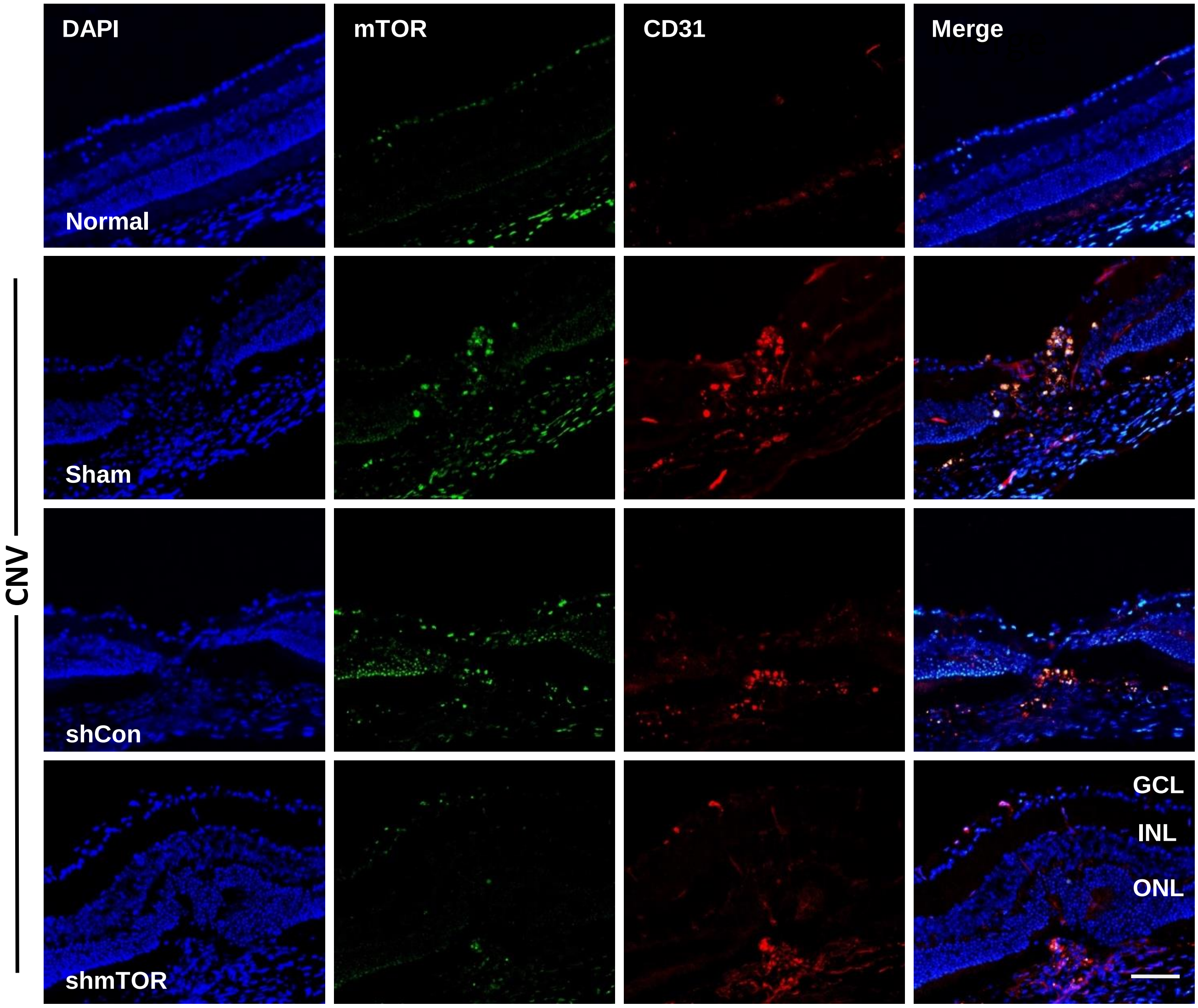

Supplemental Figure 2

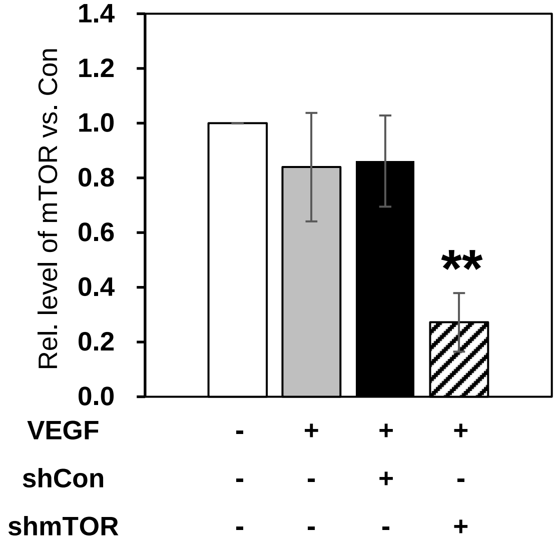

Supplemental Figure Legends

Fig. S1. Transduction of endothelial cells by AAV2 vector in the laser-induced CNV model. (A) AAV2-shCon or AAV2-shmTOR was intravitreally injected into the laser-induced photocoagulated eye of mouse. Frozen section samples were stained with anti-mTOR antibody (green) along with anti-CD31 antibody (red) to determine the retinal tissue tropism of AAV2 vectors. Nuclei were counterstained with DAPI (blue) Scale bars: 100 μm.

Fig. S2. Downregulation of mRNA levels of mTOR by AAV2-shmTOR. HUVECs were transduced by AAV2-GFP or AAV2-shmTOR for 24 h. mRNA levels of mTOR were examined by quantitative polymerase chain reaction. ANOVA and paired *t*-test. $^{**}p < 0.01$.
